# Remote ischaemic preconditioning influences the levels of acylcarnitines in vascular surgery: a randomised clinical trial

**DOI:** 10.1186/s12986-020-00495-3

**Published:** 2020-09-18

**Authors:** Teele Kasepalu, Karl Kuusik, Urmas Lepner, Joel Starkopf, Mihkel Zilmer, Jaan Eha, Mare Vähi, Jaak Kals

**Affiliations:** 1 Department of Surgery, Institute of Clinical Medicine, University of Tartu, Puusepa 8, 50406 Tartu, Estonia; 2 Department of Biochemistry, Institute of Biomedicine and Translational Medicine, Centre of Excellence for Genomics and Translational Medicine, University of Tartu, Tartu, Estonia; 3 Department of Cardiology, Institute of Clinical Medicine, University of Tartu, Tartu, Estonia; 4 Tartu University Hospital, Tartu, Estonia; 5 Department of Anaesthesiology and Intensive Care, Institute of Clinical Medicine, University of Tartu, Tartu, Estonia; 6 Institute of Mathematics and Statistics, University of Tartu, Tartu, Estonia

**Keywords:** Acylcarnitines, Ischaemic preconditioning, Mitochondria, Vascular surgery

## Abstract

**Background:**

Vascular surgery patients have reduced tissues` blood supply, which may lead to mitochondrial dysfunction and accumulation of acylcarnitines (ACs). It has been suggested that remote ischaemic preconditioning (RIPC) has its organ protective effect via promoting mitochondrial function. The aim of this study was to evaluate the effect of RIPC on the profile of ACs in the vascular surgery patients.

**Methods:**

This is a randomised, sham-controlled, double-blinded, single-centre study. Patients undergoing open surgical repair of abdominal aortic aneurysm, surgical lower limb revascularisation surgery or carotid endarterectomy were recruited non-consecutively. The RIPC protocol consisting of 4 cycles of 5 min of ischaemia, followed by 5 min of reperfusion, was applied. A blood pressure cuff was used for RIPC or a sham procedure. Blood was collected preoperatively and approximately 24 h postoperatively. The profile of ACs was analysed using the AbsoluteIDQp180 kit (Biocrates Life Sciences AG, Innsbruck, Austria).

**Results:**

Ninety-eight patients were recruited and randomised into the study groups and 45 patients from the RIPC group and 47 patients from the sham group were included in final analysis. There was a statistically significant difference between the groups regarding the changes in C3-OH (*p* = 0.023)—there was a decrease (− 0.007 µmol/L, ± 0.020 µmol/L, *p* = 0.0233) in the RIPC group and increase (0.002 µmol/L, ± 0.015 µmol/L, *p* = 0.481) in the sham group. Additionally, a decrease from baseline to 24 h after surgery (*p* < 0.05) was detected both in the sham and the RIPC group in the levels of following ACs: C2, C8, C10, C10:1, C12, C12:1, C14:1, C14:2, C16, C16:1, C18, C18:1, C18:2. In the sham group, there was an increase (*p* < 0.05) in the levels of C0 (carnitine) and a decrease in the level of C18:1-OH. In the RIPC group, a decrease (*p* < 0.05) was noted in the levels of C3-OH, C3-DC (C4-OH), C6:1, C9, C10:2.

**Conclusions:**

It can be concluded that RIPC may have an effect on the levels of ACs and might therefore have protective effects on mitochondria in the vascular surgery patients. Further larger studies conducted on homogenous populations are needed to make more definite conclusions about the effect of RIPC on the metabolism of ACs.

**Trial registration:**

ClinicalTrials.gov database, NCT02689414. Registered 24 February 2016—Retrospectively registered, https://clinicaltrials.gov/ct2/show/NCT02689414.

**Electronic supplementary material:**

The online version of this article (10.1186/s12986-020-00495-3) contains supplementary material, which is available to authorized users.

## Background

Remote ischaemic preconditioning (RIPC) is an experimental procedure in which short episodes of ischaemia are induced in order to offer organ protection to distant tissues. We along other investigators have demonstrated that RIPC has protective effects to heart, kidneys, brain and other organs in the case of ischaemia–reperfusion injury [1–5]. The exact mechanisms of RIPC are not known, but during the last decade multiple pathways and biochemical markers involved in achieving the effect of RIPC have been discovered. It has been found that cardioprotection by RIPC occurs along with improved mitochondrial function in animal studies [6, 7]. As ischaemia–reperfusion injury is known to impair mitochondrial function, the effect of RIPC might be beneficial in reducing the extent of injury. Ischaemia–reperfusion injury is inevitable in vascular surgery. Also, surgery induces acute stress response, which in turn promotes catabolic pathways including fatty-acid catabolism in beta-oxidation hereby increasing the load on mitochondria even more. Mitochondria have a principal position in energy metabolism and intensification of the beta-oxidation of fatty acids in mitochondria leads to elevated ATP production due to integrated action of the Krebs cycle and the respiratory chain. In the case of mitochondrial dysfunction, fatty acid β-oxidation is diminished, resulting in accumulation of acylcarnitines (ACs) [8, 9]. ACs are esters of l-carnitine and fatty acids and due to existence of different fatty acids [10], a large set of ACs can be produced that are generally divided into short, medium and long chain ACs (denoted as SCACs, MCACs and LCACs). For transport of fatty acid into mitochondria for beta-oxidation, the coenzyme A group is attached and afterwards displaced by carnitine forming an acylcarnitine, which is able to enter the mitochondrial matrix where it can be broken down by carnitine palmitoyl transferase II to release activated fatty acid to enter beta-oxidation [11]. It is crucial to produce LCACs as mitochondrial inner membrane is impenetrable for long chain fatty acids. Because of this, the increase of LCACs is most commonly associated with metabolic disorders such as mitochondrial dysfunction and genetic enzyme deficiencies [11]. However, as more knowledge has been gained about the role of SCACs and MCACs, it is necessary to simultaneously investigate changes of SCACs, MCACs and LCACs.

Considering all this, shifts in the ACs profile might occur in response to RIPC in patients undergoing vascular surgery. To our knowledge, no studies have been published about the effect of RIPC on the whole profile (C2–C18) of ACs.

This is a substudy within our large clinical trial conducted for evaluating the effect of RIPC on arterial stiffness and end-organ damage [1, 2, 12]. Among our secondary aims was to investigate changes in the levels of ACs. We hypothesised that RIPC may prevent the increase of the levels of ACs, ensuing from metabolic shifts in patients undergoing vascular surgery and the aim of the current study was to test this hypothesis.

## Methods

### Study groups and eligibility

This randomised double-blinded sham-controlled clinical trial was conducted at the Department of Vascular Surgery, Clinic of Surgery, at Tartu University Hospital.

Patients undergoing open surgical repair of infra-renal abdominal aortic aneurysm (AAA) or surgical lower limb revascularisation surgery (for claudication or critical limb ischaemia; common femoral artery endarterectomy, aorto(bi)femoral or femoropopliteal or femorotibial or iliofemoral bypass surgery) or carotid endarterectomy (for symptomatic or asymptomatic carotid stenosis) during the period from January 1, 2016 to February 8, 2018 were recruited non-consecutively.

Signed informed consent was obtained from each patient.

The research protocol of our study was approved by the Research Ethics Committee of the University of Tartu, and was registered in the ClinicalTrials.gov database (NCT02689414).

The following exclusion criteria were applied: age under 18 years, pregnancy, known malignancy in the past 5 years, permanent atrial fibrillation or flutter, symptomatic upper limb atherosclerosis, need for oxygen therapy at home, estimated preoperative glomerular filtration rate (eGFR) < 30 mL/min/1.73 m^2^, myocardial infarction in the past month, previous history of upper limb vein thrombosis or vascular surgery in the axillary region, and inability to follow the study regimen.

### Intervention

The RIPC protocol consisted of four 5-min episodes of ischaemia with a 5-min period of reperfusion between the episodes, which has been one of the most often used protocols in earlier studies. Ischaemia was achieved by placing a blood pressure cuff on an arm and raising cuff pressure to 200 mmHg. In case the patient’s blood pressure exceeded 180 mmHg, the cuff pressure was raised to a value that was 20 mmHg higher than systolic blood pressure. For sham group patients, cuff pressure was kept at level of venous pressure (10–20 mmHg). Intervention began along with preparation for anaesthesia in the operating theatre. Any other aspects of surgery, including anaesthesia and medication use, were not affected. The principal investigator was in charge of patient recruitment, assignment to intervention and data storage.

### Blinding

The patient, surgeon, anaesthesiologist and everyone else in the surgical team were blinded to study intervention. The scale of the manometer was kept covered. The statistician was blinded to the meaning of the group affiliation.

### Outcomes

Blood samples for analysis of ACs were collected in the morning of surgery and approximately 24 h after surgery. The last blood collection was set as close as possible to 24 h after surgery on condition that the patient had fasted for at least 3 h. Blood samples were centrifuged, serum was separated and stored in the refrigerator at − 80 °C.

The levels of ACs were analysed using the AbsoluteIDQp180 kit (Biocrates Life Sciences AG, Innsbruck, Austria). The analytical procedure was performed according to the manufacturer’s standard protocol in the laboratory of the Department of Biochemistry, University of Tartu. In brief, for targeted analysis of metabolites internal standard was pipetted onto a 96-well extraction plate and 10 µL serum was added to each well. Drainage was achieved with nitrogen and derivatisation was performed with phenylisothiocyanate. The measurements were accomplished with QTRAP 4500 (ABSciex, USA) coupled to an Agilent 1260 series HPLC (USA), using the C18 column and flow injection analysis. The vendor’s software with internal standards’ intensities was used to calculate the concentrations of ACs along with other metabolites, which are not discussed in this paper.

Blood samples for analysis of high sensitivity troponin T (hs-TnT) and N-terminal pro-brain natriuretic peptide (NT-proBNP) which were used to calculate correlations, were collected along with the samples for analysis of ACs preoperatively and approximately 24 h after surgery. The levels of cardiac biomarkers were analysed at the United Laboratories of Tartu University Hospital.

Sandwich electrochemiluminescence immunoassays (ECLIA), specifically the Elecsys troponin T high-sensitive assay as STAT version (Roche Diagnostics) and Elecsys proBNP II (Roche Diagnostics) were used according to the manufacturer’s protocol for analysis of hs-TnT and NT-proBNP.

All patients were asked about their previous and current health issues and medications; an electronic health database was also used for complete anamnesis.

### Statistical analysis

Two groups were compared using Student’s *t *test or the Wilcoxon rank-sum or Chi-squared test as appropriate. Student’s *t *test was used in baseline characteristics comparison where normal distribution was present. Wilcoxon rank-sum test was used in baseline comparison where normal distribution was not present. Because of the issue of multiple comparison, Benjamini–Hochberg procedure was used to control false discovery rate. For assessing correlations between changes in the levels of AC and cardiac biomarkers, Spearman’s correlation coefficient was employed.

*P *values under 0.05 were considered significant. Statistical analysis was performed by a qualified statistician from the University of Tartu.

As the study’s primary outcomes were parameters of arterial stiffness, calculation of sample size was based on their values [12]. For both groups, calculated sample size was 44.

## Results

Ninety-eight patients were recruited and randomised into the study groups and 45 patients from the RIPC group and 47 patients from the sham group were included in final analysis.

**Fig. 1:**
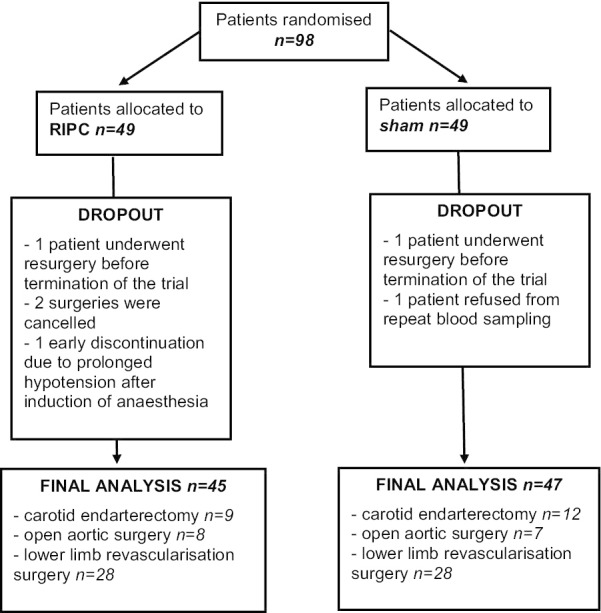
Patients’ flow chart

Detailed patient flow is depicted in Fig. 1. The median time from the end of intervention to the beginning of surgery did not differ significantly (*p* = 0.057) between the RIPC (36 min, IQR 21–46 min) and the sham group (25 min, IQR 15–38 min). There were no significant differences in the baseline values of ACs (Additional file 1). The baseline characteristics (including medications, comorbidities, preoperative risk of surgery) of the two groups were similar (Table 1). No adverse events due to RIPC were described and no patient found the RIPC or the sham procedure unbearable.

**Table 1:** Baseline characteristics

Variable	RIPC (n = 45)	SHAM (n = 47)	*p *value
Age, years (SD)	67 (± 9)	66 (± 10)	0.577
Male, n (%)	36 (80)	32 (68)	0.288
BMI, kg/m^2^ (SD)	26.3 (± 6.4)	26.5 (± 6.7)	0.840
ASA 2, n (%)	18 (40)	19 (40)	1
ASA 3, n (%)	20 (44)	22 (47)	0.986
ASA 4, n (%)	7 (16)	6 (13)	0.933
ACEI or ARB, n (%)	21 (47)	30 (64)	0.148
Calcium channel blockers, n (%)	9 (20)	17 (37)	0.135
Beta-blockers, n (%)	11 (24)	19 (40)	0.158
Statins, n (%)	13 (29)	14 (30)	1
Diabetes, n (%)	5 (11)	8 (17)	0.607
Myocardial infarction, n (%)	8 (18)	3 (6)	0.172
Stroke, n (%)	10 (22)	12 (26)	0.899
Smoker (current or ex-smoker), n (%)	40 (89)	42 (89)	1
MAP, mmHg (SD)	99 (± 12)	100 (± 11)	0.678
Heart rate, bpm (SD)	66 (± 9)	67 (± 11)	0.754
Cholesterol, mmol/L (IQR)	5.0 (4.2–5.7)	5.0 (3.9–5.6)	0.793
LDL, mmol/L (IQR)	3.4 (8.1–10.4)	3.3 (2.5–3.8)	0.500
HDL, mmol/L (IQR)	1.1 (0.9–1.4)	1.1 (1.0–1.3)	0.311
Triglycerides, mmol/L (IQR)	1.6 (1.3–1.8)	1.5 (1.2–2.0)	0.787
Administration of propofol, n (%)	19 (42)	26 (55)	0.295
Duration of surgery, min (IQR)	108 (89–135)	112 (84–156)	0.827

*BMI* body mass index, *ASA* American Society of Anaesthesiologists’ physical status score, *ACEI* angiotensin-converting-enzyme inhibitor, *ARB* angiotensin II receptor blocker, *PSBP* peripheral systolic blood pressure, *PDBP* peripheral diastolic blood pressure, *CSBP* central systolic blood pressure, *CDBP* central diastolic blood pressure, *MAP* mean arterial blood pressure, *SD* standard deviation, *IQR* interquartile range. *p *values were calculated for data with normal distribution (presented as mean and SD) with Student’s *t *test; for data with non-normal distribution (presented as median and IQR) with Wilcoxon rank-sum test; and for binary data (presented as number and percentage) with Chi-squared test

### Changes in the levels of ACs in the RIPC and in the sham group (Table 2)

**Table 2:** Changes in the levels of acylcarnitine esters from baseline to 24 h postoperatively

	SHAM		RIPC		RIPC vs SHAM* p *value
	Change (µmol/L)	*p *value	Change (µmol/L)	*p *value	
C0	2.900 (− 2.700 to 7.200)	*0.0173*	1.600 (− 2.500 to 7.200)	0.0628	0.7057
C2	− 0.850 (− 2.300 to 0.230)	*0.0149*	− 1.300 (− 2.710 to 0.270)	*0.0051*	0.7912
C3	− 0.009 (− 0.152 to 0.160)	* < 0.0001*	− 0.077 (− 0.250 to 0.083)	* < 0.0001*	0.2428
C3-DC	− 0.003 (− 0.018 to 0.010)	0.3944	− 0.004 (− 0.017 to 0.008)	*0.0488*	0.7231
C3-OH	0.002 (− 0.010 to 0.011)	0.4809	− 0.005 (− 0.018 to 0.007)	*0.0233*	*0.0232*
C3:1	− 0.002 (− 0.012 to 0.008)	0.9557	− 0.001 (− 0.014 to 0.008)	0.4578	0.6078
C4	0.008 (− 0.043 to 0.078)	* < 0.0001*	− 0.020 (− 0.075 to 0.042)	* < 0.0001*	0.2042
C4:1	0.002 (− 0.007 to 0.011)	0.2679	− 0.003 (− 0.014 to 0.011)	0.2227	0.0976
C5	− 0.005 (− 0.036 to 0.061)	* < 0.0001*	− 0.045 (− 0.107 to 0.045)	* < 0.0001*	*0.0553*
C5-DC (C6-OH)	0.003 (− 0.014 to 0.013)	0.8467	0.000 (− 0.010 to 0.017)	0.4274	0.7236
C5-M-DC	0.008 (− 0.005 to 0.026)	0.0504	0.005 (− 0.007 to 0.015)	0.1865	0.3989
C5-OH (C3-DC-M)	0.002 (− 0.013 to 0.006)	0.4681	− 0.003 (− 0.011 to 0.005)	0.3501	0.6769
C5:1	0.000 (− 0.008 to 0.010)	0.7822	− 0.007 (− 0.017 to 0.006)	0.2437	*0.0583*
C5:1-DC	0.003 (− 0.007 to 0.011)	0.0931	− 0.001 (− 0.006 to 0.007)	0.5304	0.1824
C6 (C4:1-DC)	− 0.001 (− 0.004 to 0.001)	0.2417	− 0.001 (− 0.004 to 0.001)	0.2027	0.7639
C6:1	0.000 (− 0.001 to 0.001)	0.8706	− 0.001 (− 0.001 to 0.001)	*0.0482*	0.1071
C7-DC	− 0.003 (− 0.011 to 0.007)	0.1833	− 0.001 (− 0.010 to 0.007)	0.2147	0.9025
C8	− 0.026 (− 0.062 to (− 0.001))	*0.0017*	− 0.035 (− 0.059 to 0.002)	*0.0002*	0.9658
C9	− 0.001 (− 0.014 to 0.016)	0.8637	− 0.005 (− 0.020 to 0.006)	*0.0230*	*0.0590*
C10	− 0.097 (− 0.150 to (− 0.015))	* < 0.0001*	− 0.078 (− 0.0198 to (− 0.012))	* < 0.0001*	0.9348
C10:1	− 0.022 (− 0.53 to 0.002)	*0.0005*	− 0.021 (− 0.062 to 0.001)	* < 0.0001*	0.6856
C10:2	− 0.002 (− 0.019 to 0.014)	0.4419	− 0.010 (− 0.021 to 0.002)	*0.0074*	0.1454
C12	− 0.015 (− 0.047 to 0.005)	* < 0.0001*	− 0.017 (− 0.045 to 0.006)	* < 0.0001*	0.8395
C12-DC	− 0.007 (− 0.024 to 0.005)	0.0699	0.001 (− 0.010 to 0.019)	0.9137	0.1800
C12:1	− 0.019 (− 0.045 to 0.000)	*0.0015*	− 0.030 (− 0.056 to (− 0.009))	* < 0.0001*	0.2160
C14	− 0.004 (− 0.011 to 0.003)	* < 0.0001*	− 0.004 (− 0.013 to 0.005)	* < 0.0001*	0.9039
C14:1	− 0.018 (− 0.037 to (− 0.005))	* < 0.0001*	− 0.024 (− 0.037 to (− 0.004))	* < 0.0001*	0.9379
C14:1-OH	− 0.001 (− 0.007 to 0.008)	0.8262	− 0.002 (− 0.006 to 0.004)	0.5096	0.5179
C14:2	− 0.009 (− 0.018 to (− 0.001))	* < 0.0001*	− 0.004 (− 0.015 to 0.001)	*0.0018*	0.1529
C14:2-OH	0.001 (− 0.008 to 0.007)	0.9380	0.000 (− 0.004 to 0.007)	0.2544	0.9720
C16	− 0.034 (− 0.053 to (− 0.012)	* < 0.0001*	− 0.030 (− 0.048 to (− 0.003))	* < 0.0001*	0.3668
C16-OH	− 0.004 (− 0.009 to 0.004)	0.3247	− 0.002 (− 0.014 to 0.005)	0.2893	0.7603
C16:1	− 0.012 (− 0.023 to 0.001)	* < 0.0001*	− 0.009 (− 0.019 to (− 0.002))	*0.0003*	0.6482
C16:1-OH	− 0.002 (− 0.010 to 0.005)	0.0542	− 0.004 (− 0.012 to 0.004)	0.0718	0.8257
C16:2	0.000 (− 0.009 to 0.009)	0.3098	− 0.001 (− 0.014 to 0.010)	0.4121	0.9348
C16:2-OH	− 0.002 (− 0.005 to 0.003)	0.4349	− 0.002 (− 0.006 to 0.005)	0.8756	0.8915
C18	− 0.029 (− 0.930 to (− 0.010)	* < 0.0001*	− 0.032 (− 0.860 to (− 0.010))	* < 0.0001*	0.8032
C18:1	− 0.054 (− 0.086 to (− 0.020))	* < 0.0001*	− 0.052 (− 0.082 to (− 0.026))	* < 0.0001*	0.4525
C18:1-OH	− 0.005 (− 0.014 to 0.001)	*0.0045*	0.000 (− 0.009 to 0.005)	0.9242	0.0980
C18:2	− 0.017 (− 0.024 to (− 0.004))	* < 0.0001*	− 0.016 (− 0.029 to (− 0.007))	* < 0.0001*	0.4541

Data (with non-normal distribution) is shown as median change (interquartile range). Statistically significant *p* values are in italics. *p* values were calculated with Wilcoxon rank-sum test

There was a statistically significant difference between the groups regarding the changes in C3-OH (*p* = 0.023)—there was a significant decrease (− 0.007 µmol/L, ± 0.020 µmol/L, *p* = 0.0233) in the RIPC group and insignificant increase (0.002 µmol/L, ± 0.015 µmol/L, *p* = 0.481) in the sham group.

A statistically significant decrease (*p* < 0.05) was detected both in the sham and the RIPC group in the levels of following ACs: C2, C8, C10, C10:1, C12, C12:1, C14:1, C14:2, C16, C16:1, C18, C18:1, C18:2. In the sham group, there was a statistically significant increase (*p* < 0.05) in the levels of C0 (carnitine) and a statistically significant decrease in the level of C18:1-OH. In the RIPC group, a statistically significant decrease (*p* < 0.05) was noted in the levels of C3-OH, C3-DC (C4-OH), C6:1, C9, C10:2.

### Correlations between change in hs-TnT and changes in the levels of ACs (Table 3)

**Table 3:** Substantial correlations between cardiac biomarkers (i.e. high sensitivity troponin T and NT-proBNP) and acylcarnitines

	Hs-TnT				NT-proBNP			
	RIPC (n = 45)	*p *value	SHAM (n = 47)	*p *value	RIPC (n = 45)	p*	SHAM (n = 47)	*p *value
C4	*0.38*	*0.01*	− 0.05	0.734	0.10	0.532	− 0.26	0.081
C5-DC	0.18	0.230	− 0.29	0.050	0.15	0.320	− 0.16	0.276
C5-OH	0.13	0.379	− *0.34*	*0.021*	− 0.06	0.719	0.14	0.359
C7-DC	0.07	0.639	− 0.08	0.580	0.18	0.241	0.28	0.055
C10	*0.38*	*0.010*	− 0.12	0.416	0.30	0.049	− 0.10	0.519
C10:1	*0.38*	*0.010*	− 0.12	0.404	0.18	0.225	− 0.12	0.405
C12:1	*0.31*	*0.037*	− 0.06	0.672	0.18	0.232	− 0.04	0.797
C16:1	− 0.15	0.341	− 0.25	0.090	0.10	0.500	*− 0.35*	*0.016*
C16:2	0.10	0.494	− 0.12	0.407	*0.34*	*0.021*	0.06	0.688
C18	0.05	0.745	− 0.11	0.476	0.17	0.268	*0.31*	*0.031*
C18:1	*0.32*	*0.030*	− 0.15	0.300	0.15	0.329	− 0.13	0.389
C18-OH	*0.35*	*0.019*	− 0.07	0.628	0.12	0.431	− 0.27	0.07

Statistically significant *p *values and correlations are in italics

In the RIPC group, there were statistically significant positive correlations between change of hs-TnT and change of C4 (cor = 0.38, *p* = 0.01), C10 (cor = 0.38, *p* = 0.010), C10:1 (cor = 0.38, *p* = 0.010), C12:1 (cor = 0.31, *p* = 0.037), C18:1 (cor = 0.32, *p* = 0.030) and C18-OH (cor = 0.35, *p* = 0.019). In the sham group, there was statistically significant negative correlation between change of hs-TnT and change of C5-OH (cor = − 0.34, *p* = 0.021). No other significant correlations were observed between changes in the levels of hs-TnT and ACs in the sham group.

### Correlations between change in NT-proBNP and changes in the levels of ACs (Table 3)

In the RIPC group a statistically significant positive correlation occurred between change of NT-proBNP and change of C16:2 (cor = 0.34, *p* = 0.021). In the sham group, a statistically significant positive correlation occurred between change of NT-proBNP and change of C18 (cor = 0.31, *p* = 0.031) and statistically significant negative correlation between change of NT-proBNP and change of C16:1 (cor = − 0.35, *p* = 0.016).

## Discussion

There have been no studies evaluating the effect of RIPC on the level of carnitine (C0) and on the profile of all acylcarnitines (C2–C18). In this study we describe the positive effect of RIPC in lowering the levels of several ACs in patients undergoing vascular surgery. We noted a statistically significant difference in changes in the level of C3-OH between the RIPC and sham groups. In addition, there was a statistically significant increase in the levels of C0 in the sham group, no significant increase occurred in the levels of any ACs but a statistically significant decrease occurred in the levels of C3, C3-OH, C3-DC (C4-OH), C4, C5, C6:1, C9, C10:2 in the RIPC group. All these findings indicate the RIPC-directed effect on the ACs profile in plasma.

Based on previous studies, the decrease in the levels of ACs can be associated with preserved mitochondrial function [9] whereas their increase has been linked to increased mortality in chronic heart failure patients [8] and worse prognosis in patients with IgA nephropathy [13]. Several carnitine esters and members of the ACs are elevated in patients with peripheral artery disease (PAD) [14]. Nevertheless, the impact of ACs in clinical practice is unknown as relevant studies are lacking. Considering the results of the studies published about ACs, knowledge of the ACs profile may facilitate assessment of the patients’ general metabolic milieu, mitochondrial functioning and prognosis.

ACs have a different origin in plasma. The main precursors of SCACs are branched chain amino acids (BCAAs) but some SCASs are also produced by catabolism of glucose and some triglycerides. MCACs and LCACS are only derived from fatty acid metabolism whereas carnitine is required for transporting long-chain fatty acids into mitochondria [15]. SCASs in plasma has been found to be released from the liver [16], MCACs, from the skeletal muscles and liver [17] and LCACs, from the heart [15]. Taken together, the exact origin of plasma ACs is not clear, yet based on the assessment of whole ACs spectrum, conclusions can be drawn about the whole-body acylcarnitine metabolism. We observed an increase of some SCACs levels in the sham group whereas no any increase of ACs occurred in the RIPC group. It should be noted that the hepatoprotective effect of RIPC has been reported previously [4, 18].

Evidently, the stress caused by surgery enhances the catabolism of BCCA in the liver in order to produce additional metabolic energy and this is accompanied by an increase of plasma SCACs. RIPC has been found to intensify hepatic oxygenation and hepatic microcirculation via activation of eNOS [19], as well as to increase expression of protector proteins (e.g. heme oxygenase 1) [20], which preserves mitochondrial functionality. Hence, produced SCACs can be spent more efficiently in the case of RIPC and, based on our findings we could suppose that RIPC might have a protective effect on the liver. Surgery increases the demand for metabolic energy may also cause elevated level of ketone bodies leading to production of C3-OH. In RIPC, the elimination of this SCAC is more effective compared to the sham group, which results in a statistically significant difference between these groups.

As a response to any surgery, the human organism intensifies the production of metabolic energy, which is accompanied by additional spending of long chain fatty acids for energy-rich substrates through LCACs. This can explain for the decline in LCASs both in the sham and the RIPC group.

The cardioprotective effect of RIPC has been studied on humans since 2006 [21], however, results are contradictory. Although a previously published systematic review on patients undergoing non-cardiac vascular surgery reports no effect of RIPC in reducing myocardial injury [22], its cardioprotective effect has been described in more homogenous and more powerful studies with other populations. Cardioprotection by RIPC has been associated with the improved function of mitochondria [23–25]. However, two of these studies failed to demonstrate an effect of RIPC on cardioprotection and hence also correlation between cardioprotection and salvage of mitochondrial function [23, 24].

Previously, we have published a study on the same cohort, where we demonstrated positive effects of RIPC in reducing the levels of hsTnT and NT-proBNP [2]. In the present study, we describe positive correlations between decrease of these cardiac biomarkers and decrease of several ACs in the RIPC group. In the sham group, no positive correlations were noted between increase of hsTnT and increase of ACs. On the contrary, there was negative correlation between increase of hsTnT and increase of C5-OH. Also there was a negative correlation between increase of NT-proBNP and C16:2 and positive correlation between increase of NT-proBNP and C18. The strength of correlations may have been weakened by inter-individual variations in the AC profiles coming from inter-individual differences in metabolism. Also, if our study had been larger, more definite conclusions about these correlations could be made. Although the changes of the above mentioned ACs between the groups were not significant, these differences between the sham and the RIPC group and the previously described cardioprotective effects of RIPC in the same cohort [2] suggest that the cardioprotective effects could be described basing on shifts in the levels of ACs.

However, further studies are needed to clarify these assumptions*.*

There are several limitations to our study. Firstly, the study population was heterogeneous as was also presumably their metabolic status. Although, there were no differences between study groups regarding comorbidities, health status, most common antihypertensive medications, treatment with statins and the baseline values of ACs, there might be a variation in response of ACs metabolism. It has been found that the levels of ACs differ among patients with different stages of PAD those without PAD [14]. As in our study there were patients with different locations and stages of atherosclerotic lesions, their metabolic status was evidently different, which may have influenced the results. Also we recruited both diabetic and non-diabetic patients in our groups and diabetes has been found to affect the levels of ACs [26] and have a deleterious effect on RIPC [27, 28]. Moreover, the patients underwent different types of surgery and the extent of tissue damage was different. The patients undergoing open surgical repair of AAA probably experienced larger tissue damage and were not chronically preconditioned against ischaemia like might have been PAD patients who were undergoing lower limb revascularisation surgery. Thus the type of surgery may have had a great impact on the metabolism of ACs and the effect of RIPC may have been overwhelmed. In addition, in 42% of the patients in the RIPC group propofol-induced anaesthesia was used and propofol has been found to reduce the effect of RIPC [29]. Also, the size of the study group was relatively small and the true effect of RIPC might have been missed as there were multiple differences between the groups, which did not quite reach statistical significance.

## Conclusions

It can be concluded that RIPC may have an effect on the levels of ACs and might therefore have protective effects on mitochondria in the vascular surgery patients. Further larger studies conducted on homogenous populations are needed to make more definite conclusions about the effect of RIPC on the metabolism of ACs.

## Supplementary information

**Additional file 1: Table S1**. Baseline values of acylcarnitines. **Table S2**. Non-substantial correlations between cardiac biomarkers (i.e. high sensitivity troponin T and NT-proBNP) and acylcarnitines.

## Data Availability

The datasets used and/or analysed during the current study are available from the corresponding author on reasonable request.

## Abbreviations

AAA: Abdominal aortic aneurysm
ACs: Acylcarnitines
LCACs: Long chain acylcarnitines
MSCASs: Medium chain acylcarnitines
PAD: Peripheral artery disease
RIPC: Remote ischaemic preconditioning
SCASc: Short chain acylcarnitines
