# Supplementary material for: Remote ischaemic preconditioning influences the levels of acylcarnitines in vascular surgery: a randomised clinical trial
Source: Nutr Metab (Lond). 2020 Sep 18;17:76. doi: 10.1186/s12986-020-00495-3 (PMC7501679; doi:10.1186/s12986-020-00495-3)
Supplement: Supplementary file 1 — Additional file 1: Table S1. Baseline values of acylcarnitines. Table S2. Non-substantial correlations between cardiac biomarkers (i.e. high sensitivity troponin T and NT-proBNP) and acylcarnitines. [file 12986_2020_495_MOESM1_ESM.docx]

Supplementary Table 1. Baseline values of acylcarnitines. Data are shown as median value (interquartile range).

|  | **SHAM**  **(µmol/L)** | **RIPC**  **(µmol/L)** | **RIPC vs SHAM**  **p-value** |
| --- | --- | --- | --- |
| **C0** | 45.70 (35.70−52.50) | 48.40 (43.70−55.60) | 0.1666 |
| **C2** | 8.30 (6.80−10.90) | 8.70 (6.80−10.20) | 0.8517 |
| **C3** | 2.14 (0.40−2.53) | 2.18 (0.47−2.64) | 0.3797 |
| **C3-DC** | 0.07 (0.06−0.10) | 0.07 (0.06−0.08) | 0.2979 |
| **C3-OH** | 0.07 (0.06−0.09) | 0.08 (0.05−0.10) | 0.2997 |
| **C3:1** | 0.08 (0.05−0.09) | 0.08 (0.05−0.10) | 0.7143 |
| **C4** | 0.44 (0.37−0.51) | 0.45 (0.38−0.52) | 0.6856 |
| **C4:1** | 0.08 (0.07−0.09) | 0.08 (0.07−0.09) | 0.3312 |
| **C5** | 0.70 (0.17−0.82) | 0.63 (0.21−0.83) | 0.6047 |
| **C5-DC (C6-OH)** | 0.12 (0.05−0.15) | 0.12 (0.05−0.14) | 0.5361 |
| **C5-M-DC** | 0.17(0.03−0.21) | 0.16 (0.04−0.21) | 0.9162 |
| **C5-OH (C3-DC-M)** | 0.06 (0.06−0.08) | 0.06 (0.05−0.07) | 0.1748 |
| **C5:1** | 0.08 (0.06−0.09) | 0.08 (0.06−0.10) | 0.5158 |
| **C5:1-DC** | 0.05 (0.02−0.07) | 0.06 (0.03−0.08) | 0.2651 |
| **C6 (C4:1-DC)** | 0.02 (0.01−0.02) | 0.02 (0.01−0.02) | 0.9533 |
| **C6:1** | 0.01 (0.01−0.01) | 0.01 (0.01−0.01) | 0.8715 |
| **C7-DC** | 0.05 (0.04−0.06) | 0.05 (0.04−0.06) | 0.8577 |
| **C8** | 0.18 (0.15−0.26) | 0.19 (0.16−0.27) | 0.7554 |
| **C9** | 0.08 (0.07−0.09) | 0.08 (0.07−0.09) | 0.4693 |
| **C10** | 0.34 (0.28−0.42) | 0.34 (0.25−0.43) | 0.8304 |
| **C10:1** | 0.14 (0.12−0.17) | 0.14 (0.11−0.18) | 0.8365 |
| **C10:2** | 0.10 (0.09−0.11) | 0.10 (0.09−0.11) | 0.5778 |
| **C12** | 0.13 (0.12−0.16) | 0.13 (0.12−0.15) | 0.8578 |
| **C12-DC** | 0.12 (0.10−0.19) | 0.11 (0.09−0.18) | 0.1642 |
| **C12:1** | 0.17 (0.13−0.20) | 0.16 (0.13−0.20) | 0.9751 |
| **C14** | 0.06 (0.03−0.07) | 0.05 (0.03−0.07) | 0.6543 |
| **C14:1** | 0.07 (0.05−0.10) | 0.07 (0.05−0.09) | 0.9720 |
| **C14:1-OH** | 0.03 (0.02−0.03) | 0.03 (0.02−0.04) | 0.7910 |
| **C14:2** | 0.05 (0.02−0.06) | 0.04 (0.02−0.06) | 0.4551 |
| **C14:2-OH** | 0.05 (0.02−0.05) | 0.04 (0.02−0.06) | 0.8060 |
| **C16** | 0.17 (0.13−0.21) | 0.17 (0.13−0.22) | 0.7672 |
| **C16-OH** | 0.06 (0.03−0.07) | 0.05 (0.04−0.07) | 0.8792 |
| **C16:1** | 0.08 (0.06−0.10) | 0.08 (0.06−0.10) | 0.8456 |
| **C16:1-OH** | 0.03 (0.03−0.03) | 0.03 (0.02−0.04) | 0.9565 |
| **C16:2** | 0.07 (0.06−0.09) | 0.08 (0.06−0.09) | 0.9039 |
| **C16:2-OH** | 0.03 (0.02−0.03) | 0.03 (0.02−0.03) | 0.7081 |
| **C18** | 0.09 (0.07−2.47) | 0.09 (0.07−2.53) | 0.6770 |
| **C18:1** | 0.18 (0.14−0.21) | 0.17 (0.13−0.20) | 0.4254 |
| **C18:1-OH** | 0.09 (0.04−0.10) | 0.08 (0.04−0.10) | 0.3274 |
| **C18:2** | 0.08 (0.07−0.10) | 0.09 (0.07−0.10) | 0.7732 |

*Supplementary table 2. Non-substantial correlations between cardiac biomarkers (i.e. high sensitivity troponin T and NT-proBNP) and acylcarnitines*

|  | **Hs-TnT** | | | | **NT-proBNP** | | | | | |
| --- | --- | --- | --- | --- | --- | --- | --- | --- | --- | --- |
|  | **RIPC**  **(n=45)** | **p-value** | **SHAM (n=47)** | **p-value** | **RIPC**  **(n=45)** | **p*** | **SHAM**  **(n=47)** | **p-value** | | |
|  |  |  |  |  |  |  |  |  | | |
| **C0** | -0.08 | 0.608 | -0.13 | 0.360 | 0.10 | 0.516 | 0.01 | 0.928 |  |  |
| **C2** | 0.20 | 0.190 | -0.07 | 0.638 | 0.03 | 0.855 | 0.18 | 0.236 |  |  |
| **C3** | 0.20 | 0.184 | 0.20 | 0.18 | -0.08 | 0.608 | -0.04 | 0.765 |  |  |
| **C3-DC** | 0.08 | 0.613 | -0.01 | 0.935 | 0.13 | 0.411 | 0.06 | 0.748 | |  |
| **C3-OH** | 0.11 | 0.467 | <0.01 | 0.983 | -0.06 | 0.705 | -0.01 | 0.958 | |  |
| **C3:1** | 0.06 | 0.706 | 0.05 | 0.730 | 0.18 | 0.246 | -0.16 | 0.283 | |  |
| **C4:1** | 0.15 | 0.322 | -0.04 | 0.764 | 0.18 | 0.230 | -0.03 | 0.844 | |  |
| **C5** | 0.21 | 0.172 | -0.07 | 0.630 | 0.04 | 0.770 | 0.07 | 0.621 | |  |
| **C5-M-DC** | -0.08 | 0.588 | -0.10 | 0.489 | <0.01 | 0.976 | -0.05 | 0.739 | |  |
| **C5:1** | -0.14 | 0.356 | -0.02 | 0.874 | 0.13 | 0.403 | -0.13 | 0.402 | |  |
| **C5:1-DC** | 0.07 | 0.647 | -0.14 | 0.352 | 0.15 | 0.318 | -0.19 | 0.208 | |  |
| **C6** | 0.24 | 0.119 | 0.04 | 0.788 | -0.05 | 0.725 | -0.01 | 0.960 | |  |
| **C6:1** | 0.19 | 0.206 | 0.08 | 0.581 | 0.16 | 0.309 | -0.02 | 0.909 | |  |
| **C8** | *0.26* | *0.087* | -0.15 | 0.323 | 0.24 | 0.112 | 0.05 | 0.754 | |  |
| **C9** | 0.26 | 0.086 | -0.01 | 0.964 | 0.09 | 0.575 | -0.02 | 0.904 | |  |
| **C10:2** | 0.04 | 0.795 | -0.08 | 0.595 | 0.08 | 0.587 | 0.06 | 0.667 | |  |
| **C12** | 0.17 | 0.272 | 0.05 | 0.754 | 0.02 | 0.888 | 0.08 | 0.603 | |  |
| **12-DC** | -0.07 | 0.644 | -0.10 | 0.50 | -0.06 | 0.719 | -0.17 | 0.251 | |  |
| **C14** | 0.06 | 0.691 | -0.02 | 0.915 | *0.25* | *0.100* | 0.03 | 0.824 | |  |
| **C14:1** | 0.14 | 0.349 | -0.11 | 0.444 | 0.15 | 0.311 | -0.05 | 0.741 | |  |
| **C14:1-OH** | 0.10 | 0.531 | -0.19 | 0.207 | *0.25* | *0.100* | -0.208 | 0.160 | |  |
| **C14:2** | 0.22 | 0.140 | -0.07 | 0.656 | 0.13 | 0.405 | -0.23 | 0.120 | |  |
| **C14:2-OH** | -0.15 | 0.327 | -0.09 | 0.559 | -0.09 | 0.552 | -0.05 | 0.757 | |  |
| **C16** | -0.11 | 0.478 | -0.08 | 0.584 | 0.06 | 0.691 | -0.03 | 0.855 | |  |
| **C16-OH** | -0.22 | 0.147 | 0.10 | 0.516 | 0.09 | 0.554 | 0.08 | 0.587 | |  |
| **C16:1-OH** | -0.10 | 0.521 | 0.24 | 0.112 | 0.19 | 0.217 | 0.14 | 0.364 | |  |
| **C16:2-OH** | 0.12 | 0.451 | -0.13 | 0.370 | 0.13 | 0.396 | -0.02 | 0.909 | |  |
| **C18:2** | 0.16 | 0.297 | <0.01 | 0.995 | 0.14 | 0.359 | -0.04 | 0.785 | |  |
